# Supplementary material for: Effectiveness Evaluation of a UV-C-Photoinactivator against Selected ESKAPE-E Pathogens
Source: Int J Environ Res Public Health. 2022 Dec 9;19(24):16559. doi: 10.3390/ijerph192416559 (PMC9778679; doi:10.3390/ijerph192416559)
Supplement: Supplementary file 1 [file ijerph-19-16559-s001.zip › ijerph-2047262-supplementary.pdf]

Effectiveness Evaluation of a UV-C-Photoinactivator against Selected ESKAPE-E Pathogens

Karyne Rangel [1,2,*], Fellipe O. Cabral [3], Guilherme C. Lechuga [1,2], Maria H. S. Villas-Bôas [4], Victor Midlej [5] and Salvatore G. De-Simone [1,2,6,*]

[1] Center for Technological Development in Health (CDTS)/National Institute of Science and Technology for Innovation in Neglected Population Diseases (INCT-IDPN), Oswaldo Cruz Foundation (FIOCRUZ), Rio de Janeiro 21040-900, RJ, Brazil
[2] Laboratory of Epidemiology and Molecular Systematics (LESM), Oswaldo Cruz Institute, Oswaldo Cruz Foundation (FIOCRUZ), Rio de Janeiro 21040-900, RJ, Brazil
[3] Health Sciences Center, Institute of Microbiology Paulo de Góes, Federal University of Rio de Janeiro (UFRJ), Rio de Janeiro 21941-853, RJ, Brazil
[4] Microbiology Department, National Institute for Quality Control in Health (INCQS), Oswaldo Cruz Foundation (FIOCRUZ), Rio de Janeiro 21040-900, RJ, Brazil
[5] Structural Biology Laboratory (LBE), Oswaldo Cruz Institute, Oswaldo Cruz Foundation (FIOCRUZ), Rio de Janeiro 21040-900, RJ, Brazil
[6] Post-Graduation Program in Science and Biotechnology, Department of Molecular and Cellular Biology, Biology Institute, Federal Fluminense University (UFF), Niterói 22040-036, RJ, Brazil
* Correspondence: karyne.rangelk@gmail.com (K.R.); salvatore.simone@fiocruz.br (S.G.D.-S.); Tel.: +55-21386581-82 (K.R. & S.G.D.-S.)

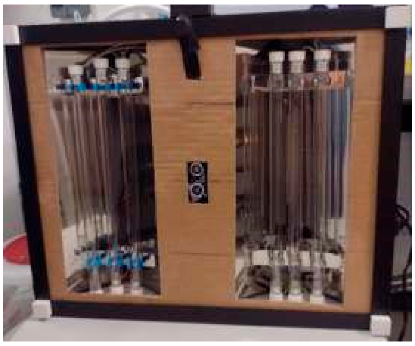

Figure S1. Front view of the upper platform of the Sanitech UVC shoe sole decontaminator equipment.

	1	2	3	4	5	6	7	8	9	10	11	12
A												
B	Sa	Pa	Se	Ec	Ab	Ef				PBS		LB
C												
D												
E												
F												
G												
H												

Figure S2. Arrangement of ATCC bacterial strains in the microplate for exposure to the UVC system and control group (no treatment). Abbreviations: Sa, *S. aureus* (ATCC 6538); Pa, *P. aeruginosa* (ATCC 15442); Se, *S. enterica* (ATCC 10708); Ec, *E. coli* (ATCC 25922); Ab, *A. baumannii* (ATCC 19606); Ef, *E. faecalis* (ATCC 29212); PBS, sterile phosphate buffered saline; L, Luria Bertani broth.
